# Pilot study of a comprehensive resource estimation method from environmental DNA using universal D-loop amplification primers

**DOI:** 10.1007/s10142-023-01013-3

**Published:** 2023-03-22

**Authors:** Kazutoshi Yoshitake, Kyohei Yanagisawa, Yuma Sugimoto, Hiroshi Nakamura, Nanami Mizusawa, Masaki Miya, Koji Hamasaki, Takanori Kobayashi, Shugo Watabe, Kazuomi Nishikiori, Shuichi Asakawa

**Affiliations:** 1 Department of Aquatic Bioscience, Graduate School of Agricultural and Life Sciences, The University of Tokyo, 1-1-1 Yayoi, Bunkyo-ku, 113-8657 Tokyo, Japan; 2 Tokyo Sea Life Park, 6-2-3 Rinkai-cho, Edogawa-ku, 134-8587 Tokyo, Japan; 3 School of Marine Biosciences, Kitasato University, 1-15-1 Kitasato, Minami-ku, Kanagawa 252-0373 Sagamihara, Japan; 4 Department of Collection Management, Natural History Museum and Institute, Chiba, 260-8682 Japan; 5 Department of Marine Ecosystem Science, Atmosphere and Ocean Research Institute, The University of Tokyo, 5-1-5 Kashiwanoha, Kashiwa, Chiba, 277-8564 Japan; 6 Department of Integrated Biosciences, Graduate School of Frontier Sciences, The University of Tokyo, 5-1-5 Kashiwanoha, Kashiwa, Chiba, 277-8564 Japan; 7 Collaborative Research Institute for Innovative Microbiology, The University of Tokyo, 1-1-1 Yayoi, Bunkyo-ku, Tokyo, 113-8657 Japan

**Keywords:** HaCeD-Seq, D-loop haplotype, Fish resource estimation, Nanopore with UMI

## Abstract

Many studies have investigated the ability of environmental DNA (eDNA) to identify the species. However, when individual species are to be identified, accurate estimation of their abundance using traditional eDNA analyses is still difficult. We previously developed a novel analytical method called HaCeD-Seq (haplotype count from eDNA by sequencing), which focuses on the mitochondrial D-loop sequence for eels and tuna. In this study, universal D-loop primers were designed to enable the comprehensive detection of multiple fish species by a single sequence. To sequence the full-length D-loop with high accuracy, we performed nanopore sequencing with unique molecular identifiers (UMI). In addition, to determine the D-loop reference sequence, whole genome sequencing was performed with thin coverage, and complete mitochondrial genomes were determined. We developed a UMI-based Nanopore D-loop sequencing analysis pipeline and released it as open-source software. We detected 5 out of 15 species (33%) and 10 haplotypes out of 35 individuals (29%) among the detected species. This study demonstrates the possibility of comprehensively obtaining information related to population size from eDNA. In the future, this method can be used to improve the accuracy of fish resource estimation, which is currently highly dependent on fishing catches.

**Supplementary Information:**

The online version contains supplementary material available at 10.1007/s10142-023-01013-3.

## Introduction

The advent of high-throughput sequencing has enabled the assessment of biodiversity in the environment using the genetic information contained in environmental DNA (eDNA) in water, soil, and air (Rees et al. 2014; Thomsen and Willerslev 2015; Ruppert et al. 2019; Garlapati et al. 2019; Clare et al. 2021). Many studies have estimated abundance using eDNA (Knudsen et al. 2019; Salter et al. 2019; Stoeckle et al. 2020). However, biomass estimation using previously reported eDNA had some limitations. One of the major problems is that different quantities of cells are released into the environment from different individuals, even in the same species and in the same environment, due to variations in size and age-class distributions (Maruyama et al. 2014) as well as alterations in environmental factors such as water temperature and flow (Jane et al. 2015). We recently developed a novel method for the reliable and simple estimation of fish population abundance from eDNA using the D-loop haplotype count for Anguillids (Yoshitake et al. 2019) and Pacific bluefin tuna *Thunnus orientalis* (Yoshitake et al. 2021). This method, called HaCeD (haplotype count from eDNA)-Seq, was successfully adopted to noninvasively estimate the abundances of various eels or tunas inhabiting the same experimental tank using eDNA. There were several problems for conventional eDNA survey methods with 12S rRNA because the different quantities of cells are released into water due to variations in size and age-class distributions (Maruyama et al. 2014) as well as alteration in environmental factors such as water temperature and flow (Jane et al. 2015). On the other hand, HaCeD-Seq is a method that can directly measure the number of individuals in eDNA, taking advantage of the fact that the D-loop sequence differs from one individual to another in eels and tuna.

HaCeD-Seq is not limited to eels and tuna, but can be applied to any organism with high mitochondrial D-loop diversity. High haplotype diversity of D-loop has already been reported for some commercially important small pelagic fish such as jack mackerel (0.964–1.000) (Song et al. 2013), chub mackerel (0.505–0.967) (Zhu et al. 2016), and spotted mackerel (0.996) (Tzeng 2007) for which the total allowable catch (TAC) has been set in the fishery management system of Japan (Ichinokawa et al. 2017). The haplotype diversity of salmon *Oncorhynchus masou* returning to their home rivers is low (Okabe et al. 2020), but the haplotype diversity of many marine fishes is high (Sang et al. 1994; Ishikawa et al. 2001; Tzeng 2007; Song et al. 2013; Nomura et al. 2014; Zhu et al. 2016; Kumar et al. 2016).

HaCeD-Seq could be used for stock assessments of above major target species with high haplotype diversity in fisheries. However, unresolved issues remain. One problem is that primers for amplifying the D-loop are species-specific. Because it is laborious to design primers and count the D-loop haplotypes for each target species, we aimed to design generic D-loop primers. In addition, although the 12S rRNA gene sequences of many species have been sequenced and registered in public databases for metabarcoding analysis, such as MiFish primers (Miya et al. 2020), D-loop sequences have not sufficiently been sequenced. It is necessary to feed databanks with D-loop sequences as reference data. In this study, we efficiently sequenced whole mitochondrial genomes, including the D-loop, as reference sequences and registered the D-loop sequence in the GenBank database. Although universal D-loop primers designed based on D-loops from 161 fish species have been reported (Cheng et al. 2012), we redesigned universal primers for the D-loop based on complete mitochondrial sequences of 2854 fish species and validated a method to obtain population information for multiple fish species.

## Materials and methods

### Universal D-loop primer design

We downloaded the MitoFish database (2020/2/20) (Iwasaki et al. 2013), containing 2854 species of complete mitochondrial sequences. The D-loop sequence and the surrounding 1000 bp sequence of the Japanese eel *Anguilla japonica* (Accession ID: AB038556) were extracted, and a homology search was performed using blastn (2.6.0 +) (Camacho et al. 2009) to MitoFish database with default parameters. Sequences of the hit regions were extracted, and multiple alignments were performed using Mafft (v7.402) (Katoh et al. 2002). The consensus sequences of the positions that were aligned in more than 90% of the species were extracted, and the percent identity of 2854 species and the consensus sequences was calculated. Since a simple percent identity for each position did not tell us whether the region was continuously conserved or not, we calculated the gap-considered identity score by subtracting the bases in the region that were aligned less than 90% of the species from the identity bases following the gap. Multiple alignments were displayed in Geneious Prime 2020.1 (Biomatters), and highly conserved regions were extracted. Sites with less than 85% conservation were designed as mixed bases in the primers. Sequence conservation was visualized using WebLogo (Crooks et al. 2004). To test the designed primers in silico, primer sequences covering all patterns of mixed bases were prepared in a FASTA file, and blastn was performed with the options of word_size 4 and max_target_seqs 10,000 against the MitoFish database.

### Library preparation and sequencing for mitochondria

We decided to sequence mitochondria from 45 species of fish kept at Tokyo Sea Life Park and whose DNA is preserved at the Chiba Prefectural Museum. DNA concentrations were quantified by electrophoresis and absorbance, and DNA extracts were obtained from 45 fish (Table 1) and mixed to achieve equal DNA concentrations. After library preparation using the Nextera DNA Flex Library Preparation Kit (Illumina), one-lane sequencing was performed using a HiSeqX sequencer at Macrogen (Seoul, Korea).

**Table 1:** List of 45 fish species sequenced in this study. A list of fish species whose mitochondria were sequenced in this study and their GenBank accession numbers is shown

Species name	Past reports	The quality of our mitochondrial genome	GenBank accession numbers
*Omobranchus punctatus*	Partial	***	OK554507
*Parupeneus ciliatus*	Partial	***	OK554512
*Chaetodon argentatus*	Partial	***	OK554514
*Stethojulis interrupta*	Partial	***	OK554515
*Coris aygula*	Partial	***	OK554516
*Parapriacanthus ransonneti*	Partial	***	OK554518
*Oxycirrhites typus*	Partial	***	OK554521
*Canthigaster axiologus*	Partial	***	OK554526
*Cirrhitops hubbardi*	Partial	***	OK554529
*Periophthalmus modestus*	Partial	***	OK554533
*Thalassoma cupido*	Partial	***	OK554535
*Istiblennius edentulus*	Partial	***	OK554536
*Nuchequula nuchalis*	Partial	***	OK554537
*Platycephalus indicus*	Partial	***	OK554543
*Pterois lunulata*	Partial	***	OK554544
*Urocampus nanus*	Partial	*	OK554510
*Evistias acutirostris*	Partial	*	OK554520
*Prionurus scalprum*	Partial	*	OK554534
*Alectis ciliaris*	Complete	***	OK554508
*Parupeneus multifasciatus*	Complete	***	OK554511
*Scarus schlegeli*	Complete	***	OK554513
*Pseudanthias squamipinnis*	Complete	***	OK554517
*Kuhlia mugil*	Complete	***	OK554519
*Konosirus punctatus*	Complete	***	OK554522
*Sillago japonica*	Complete	***	OK554524
*Lateolabrax japonicus*	Complete	***	OK554527
*Labracoglossa argentiventris*	Complete	***	OK554530
*Chelidonichthys spinosus*	Complete	***	OK554538
*Mugil cephalus*	Complete	***	OK554539
*Conger myriaster*	Complete	***	OK554541
*Etrumeus micropus*	Complete	***	OK554542
*Rudarius ercodes*	Complete	**	OK554506
*Psenopsis anomala*	Complete	**	OK554509
*Plotosus japonicus*	Complete	**	OK554523
*Pennahia argentata*	Complete	**	OK554525
*Chromis notata*	Complete	**	OK554528
*Pomacanthus imperator*	Complete	**	OK554531
*Zanclus cornutus*	Complete	**	OK554532
*Trachurus japonicus*	Complete	**	OK554540
*Centropyge interruptus*	Complete	*	OK554545
*Triacanthus biaculeatus*	Complete	***	
*Parajulis poecilepterus*	Complete	**	
*Pterocaesio tile*	Complete	**	
*Chaetodon auripes*	Complete	**	
*Halichoeres tenuispinis*	Complete	**	

***Completed

**completed (combined with past reports)

*non-completed

### Mitochondrial full-length sequence analysis

Whole genome shotgun sequencing data from a mixture of 45 species were assembled using CLC Genomics Workbench 8 (QIAGEN) and Megahit v1.1.1 (Li et al. 2015), respectively. The assembled contigs were subjected to a BLASTN homology search against MitoFish, and contigs with > 70% homology and > 10 kb length were extracted. Extracted contigs were annotated using MitoFish Annotator (Iwasaki et al. 2013) to remove noisy contigs without 12S rRNA and D-loop. Subsequently, the contigs were compared with the MitoFish and NCBI nt databases to determine the species of the contigs based on some of the registered information, such as 12S rRNA, and the mitochondrial sequences were cleaned up manually by removing chimeras. A total of 45 mitochondrial sequences were obtained and registered in GenBank except for five that were almost exact matches to known sequences (accession numbers: OK554506-OK554545).

### Water sampling and eDNA extraction

Two liters of water was collected from tank 36 at Tokyo Sea Life Park and filtered through a 0.45 µm Sterivex filter (Merck) following the eDNA Society Manual (https://ednasociety.org/en/manuals/). Briefly, after filtering the water with a syringe manually, the filter was stored with RNAlter (Thermo Fisher), and the next day, after removing the RNAlter, 200 µL of Buffer AL of Dneasy Blood and Tissue kit (QIAGEN), 220 µL of phosphate-buffered saline (PBS)(–), and 20 µL of proteinase K (20 mg/mL) were added. The mixture was sealed with Parafilm and rotated at 56 °C for 20 min. The inlet of the Sterivex filter was inserted into a 2.0-mL tube and centrifuged in a 50-mL tube at 6000 g for 1 min. The eluted solution was added with 200 µL of 100% ethanol and transferred to a column. After centrifugation at 6000 g for 1 min, 500 µL of Buffer AW1 was added to the column and centrifuged at 6000 g for 1 min. Next, 500 µL of Buffer AW2 was added to the column, which was centrifuged at 2000 × g for 3 min. Finally, the column was added with 200 µL of Buffer AE, which was left at room temperature for 1 min, centrifuged at 6000 × g for 1 min, and eDNA was collected.

### Amplification of D-loop region from eDNA for Nanopore sequencing

The adapter design of the primers for Nanopore sequencing with UMI followed the previous paper (Karst et al. 2021), adding N13 to both ends of forward and reverse primers. The first PCR to introduce the unique molecular identifiers (UMI) was performed under the following conditions: We mixed 10 µL of repliQa HiFi ToughMix (Quantabio), 0.6 µL of 10 µM forward primer (D-loop-F-UMI: CTCTTCCGATCTGTCNNNNNNNNNNNNNARAGCRYCGGTCTTGTAA), 0.6 µL of 10 µM reverse primer (D-loop-R-UMI: CTCTTCCGATCTCAGNNNNNNNNNNNNNCGGAKACTTGCATGTRTAA), 1 µL of template DNA, and 7.8 µL of nuclease-free water for a total of 20-µL reaction mixture. After denaturation at 95 °C for 3 min, five cycles of amplification were carried out at 98 °C for 10 s, 40 °C for 5 s, and 68 °C for 5 s, followed by extension at 68 °C for 1 min. The samples were then loaded onto 1.7% agarose gel and subjected to electrophoresis at 50 V for 30 min. The gel mold for electrophoresis was printed using an ELEGOO MARS PRO 3D printer (Elegoo, Shenzhen, China) with the model of Supplementary Information 1, and 3D printer resin SK01F (SK HONPO, Tokyo, Japan). The 3D model was designed using a Fusion 360 (Autodesk). A disposable electrophoresis chamber was created to prevent contamination (Supplementary Fig. 1). A disposable electrophoresis chamber was made using knock-off pencil refills 2.0 HB (Takumi, Tokyo, Japan) and a silicon lunch square cup C-4974 (PEARL METAL, Tokyo, Japan). After electrophoresis, the gel with the size of 800–1500 bp was cut out and DNA was purified using the FastGene Gel/PCR Extraction Kit (NIPPON Genetics) and extracted with 20 µL of nuclease-free water.

A second PCR to amplify the D-loop with UMI was performed under the following conditions: We mixed 10 µL of repliQa HiFi ToughMix, 0.6 µL of10 µM forward primer (UMI-2nd-F: TACACGACGCTCTTCCGATCTGTC), 0.6 µL of 10 µM reverse primer (UMI-2nd-R: AGACGTGTGCTCTTCCGATCTCAG), 2 µL of template DNA, and 6.8 µL of nuclease-free water for a total of 20 µL. After denaturation at 95 °C for 3 min, 45 cycles of amplification were carried out at 98 °C for 10 s, 40 °C for 5 s, and 68 °C for 5 s, followed by extension at 68 °C for 1 min. The PCR product was then loaded onto a 1.7% agarose gel in a disposable electrophoresis chamber, and after electrophoresis at 50 V for 30 min, the band with a size of 800–1500 bp was cut out and purified using a FastGene Gel/PCR Extraction Kit with the elution of 20 µL of nuclease-free water.

### Sequencing with Oxford Nanopore

Nanopore sequencing was performed using a MinION flow cell R10.3 and Ligation kit SQK-LSK110 (Oxford Nanopore Technologies). Fast5 files were created by Nanopore, transferred to a Linux computer with GPU, and basecalled using Guppy v5.0.11 (Oxford Nanopore Technologies) with the super-accuracy basecalling model.

### Amplification of D-loop region from eDNA for LoopSeq

Using the same eDNA samples sequenced by Nanopore, we mixed 0.2 µL of Ex Taq (5 U/µl) (Takara), 2 µL of 10 × Ex Taq Buffer, 1.6 µL of 2.5 mM dNTP Mixture, 0.5 µL of Template DNA, 1 µL of 10 µM forward primer (D-loop-F-UMI), 1 µL of 10 µM reverse primer (D-loop-R-UMI), and nuclease-free water up to 20 µL. After denaturation at 94 °C for 2 min, 40 cycles of amplification were carried out at 94 °C for 30 s, 50 °C annealing temperature for 30 s, and 72 °C for 80 s, followed by extension at 72 °C for 5 min. The PCR product was then loaded onto a 1.7% agarose gel, and after electrophoresis, the band with a size of 1000–1500 bp was cut out and purified using the FastGene Gel/PCR Extraction Kit with the elution of 20 µL of nuclease-free water. LoopSeq libraries were prepared using the LoopSeq PCR Amplicon 24 sample Kit (Loop Genomics) and sequenced with HiSeqX 1 lane at Macrogen. Sequenced data were uploaded to the LoopSeq website, and synthetic long reads were generated.

### Universal HaCeD-Seq analysis

Only Nanopore reads that retained at least 80% homology were extracted from sequences that annealed around the D-loop region of the 1st PCR primer and the 2nd PCR primer sequence. If the UMI tag regions matched more than 80% identities with the more abundant tags, they were grouped as originated from the more abundant UMI. If a UMI pair shared one forward or reverse UMI with another UMI pair and had a different UMI on the opposite side, the UMI pair with the highest number of reads was adopted, and the other UMI pairs were removed as UMI chimeras. One hundred reads were extracted for each extracted UMI pair, and multiple alignments were performed using MUSCLE (Edgar 2004) to create consensus D-loop sequences. Using these consensus D-loop sequences and all Nanopore reads, we created a more accurate consensus D-loop sequence using MEDAKA v1.3.2 (https://github.com/nanoporetech/medaka). We also created a BLAST database by adding our additional 45 mitochondrial sequences to the MitoFish database (2020/2/20) (Iwasaki et al. 2013) and performed blastn on the consensus D-loop sequences to determine species names. Since MEDAKA did not appear to completely remove errors, we decided to check whether the haplotypes that were polished as different haplotypes were really different haplotypes. When reads derived from a haplotype are compared to another haplotype, the mutation should be called if the haplotype is really different. Mutations were called by MEDAKA_Haploid_variant using the haplotype of the cluster with the highest number of reads for each species as the reference genome, and the reads of haplotypes with smaller clusters for correction. If no mutation was called for the reads from a small cluster, the actual haplotype of the small cluster was considered identical to the highest one, so the haplotypes for which no mutation was called were removed as noise. Once the calculation was performed for the haplotype of the cluster with the highest number of reads, the process was repeated for the haplotype with the largest number of reads among the unmerged haplotypes. Finally, the number of haplotypes was calculated for each species. All the above steps were published as the eDNA ~ HaCeD-Seq_with_UMI_using_Nanopore script in the Portable Pipeline (https://github.com/c2997108/OpenPortablePipeline/blob/master/PortablePipeline/scripts/eDNA~HaCeD-Seq_with_UMI_using_Nanopore).

## Results

### Design of universal primers

**Fig. 1:**
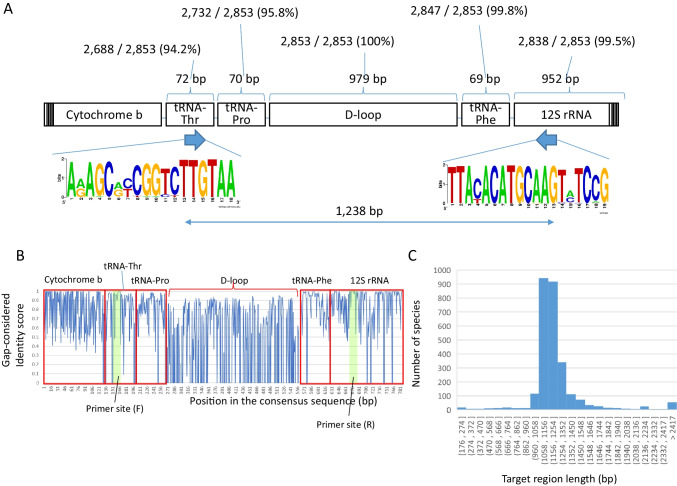
Gene structures around fish mitochondrial D-loop. **A** Schematic of fish mitochondria around the D-loop was created based on the annotation information of 2853 species registered in MitoFish database. The D-loop is annotated in all species in MitoFish database. The ratio percentage of tRNA-Pro adjacent to the D-loop in 2853 fish species is 95.8%, the percentage of tRNA-Thr adjacent to tRNA-Pro or D-loops is 94.2%, the percentage of tRNA-Phe adjacent to opposite site of the D-loop is 99.8%, and the percentage of 12S rRNA adjacent to tRNA-Phe or D-loops is 99.5%. The length of each gene represents the average length. **B** Gap-considered identity scores around the D-loop. **C** Distribution of the lengths between forward and reverse primers of 2,786 mitochondrial sequences

**Fig. 2:**
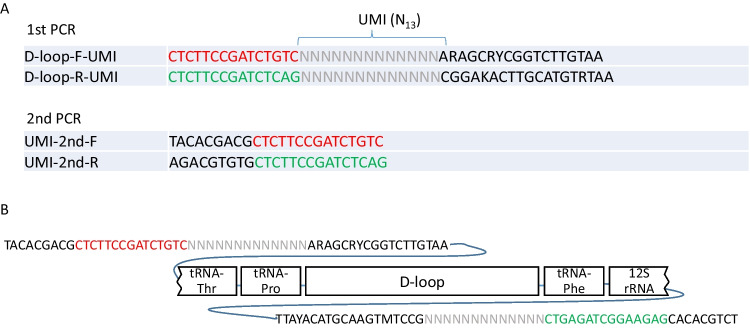
Universal HaCeD-Seq primers. **A** Primer sequence designed for the highly conserved regions in the tRNA-Thr and 12S rRNA genes next to the D-loop, linked to N13 for UMI and an adapter for 2nd PCR. 2nd PCR binds to the red and green parts of the 1st PCR primer, respectively. **B** Schematic of the final PCR product. UMI tags are inserted on both sides of forward and reverse

D-loop sequences were extracted from 2854 full-length mitochondria registered in the MitoFish database (Iwasaki et al. 2013), and multiple alignments were generated. The tRNA-Thr, tRNA-Phe, and 12S rRNA were the most highly conserved and existing regions at both ends of the D-loop (Fig. 1A, B). To compare which region is more conserved between tRNA-Phe (Fig. 1B, 579–598 bp) and 12S rRNA (Fig. 1B, 671–689 bp), we designed tRNA-Phe-rev: CATCTTCAGTGYYATGCTTT as a primer candidate sequence for tRNA-Phe region and 12S rRNA-rev: CGGAKACTTGCATGTRTAA as a primer candidate sequence for 12S rRNA region. The results of blast search considering the degenerate bases of each primer showed that 2747 (96.3%) of 2853 species were hit by tRNA-Phe-rev, while 2817 (98.7%) of 2853 species were hit by 12S-rRNA-rev. Therefore, we decided to adopt the 12S-rRNA-rev sequence. Primer sequences were designed for each of these highly conserved together with existing regions (Fig. 2A), with N13 as the UMI tag and adapter sequence for the 2nd PCR. The average length of the amplified DNA fragments was 1238 bp, the length of the lower 25% was 1107 bp, and the length of the upper 25% was 1261 bp (Fig. 1C). A schematic representation of the final PCR products after 2nd PCR is shown in Fig. 2B.

### Construction of mitochondrial reference sequences

The 12S rRNA region in mitochondria is often partially sequenced, and there are many reference sequences, but validated D-loop reference sequences are currently insufficient to support species assignment. The copy number of mitochondria is tens to hundreds of times greater than that of the nuclear genome (Hartmann et al. 2011; Fan et al. 2022), and full-length mitochondria can be easily assembled by low-coverage shotgun whole genome sequencing. Therefore, we attempted to add the D-loop sequence of fish species living in the seas around Japan to the database by sequencing the complete mitochondrial genome using thin shotgun whole-genome sequencing (Table 1). As a result, we were able to determine mitochondrial genome sequences of more than 10 kb, including the whole D-loop for 45 species. Although only partial sequences of the mitochondrial genome were registered in the public database for 18 of the 45 species and D-loop sequences were unknown, we were able to determine D-loop sequences with our sequencing data. We registered 40 mitochondrial sequences that were novel or had many polymorphisms compared to the GenBank database, excluding five species for which the sequences were nearly identical to known sequences (Accession ID: OK554506-OK554545).

### PCR and sequencing of D-loop

**Fig. 3:**
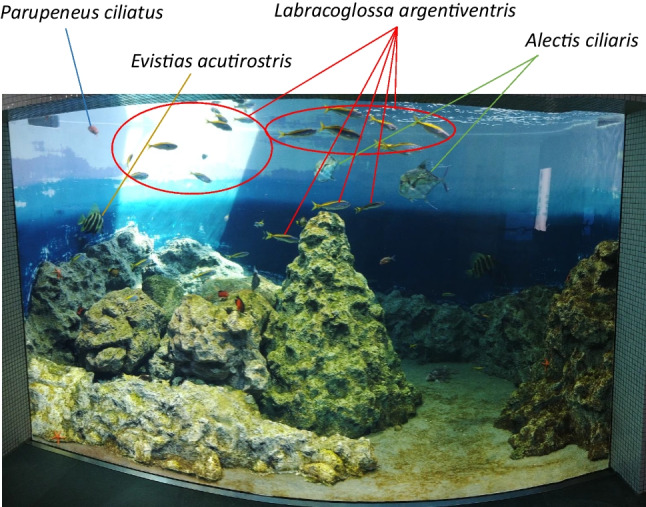
Photo of Tank No. 36 of Tokyo Sea Life Park. The filtration water flowed from the upper right of the tank. Water was collected from the upper-left side of the tank using a bucket. *Parupeneus ciliatus*, *Evistias acutirostris*, *Labracoglossa argentiventris*, and *Alectis ciliaris* swim around the surface of the tank

**Fig. 4:**
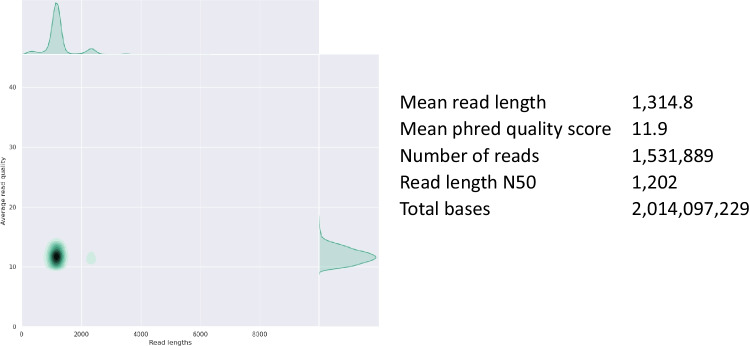
Read length and quality distribution. The read length and quality distribution of the sequenced Nanopore reads are shown. This graph was drawn using NanoPlot version 1.32.1

To evaluate the designed primers, we conducted a study in tank 36 of Tokyo Sea Life Park, where 14 species of fish were kept (Fig. 3). A two-step PCR was performed to add UMI to eDNA extracted from water in tank 36, resulting in a broad band around 1.2 kbp (Supplementary Fig. 2A). PCR products were sequenced using a Nanopore MinION flow cell. The sequenced nanopore reads contained 1,533,199 reads. The obtained read-length and quality distributions are shown in Fig. 4. In addition to Nanopore, we used LoopSeq, which is capable of synthetic long-read sequencing using Illumina, for validation. The use of custom UMI tags is not recommended for LoopSeq because the library conditioning kit for LoopSeq uses another UMI. Therefore, we performed a normal one-step PCR without UMI for LoopSeq and obtained a band around 1.2 kbp (Supplementary Fig. 2B). The PCR product was sequenced for the full-length D-loop using a LoopSeq library preparation kit. The reads obtained were 50,663 reads.

### Data analysis

**Fig. 5:**
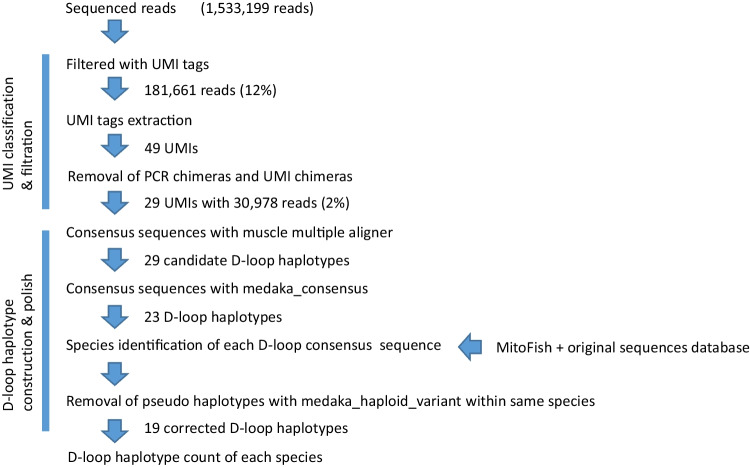
The analysis flow of the Nanopore sequenced reads. The flow of the nanopore read analysis is presented. Briefly, UMI tags were searched, reads with identical UMIs were gathered for error correction, and the number of haplotypes obtained for each species was counted

The analysis flow of nanopore-sequenced reads is shown in Fig. 5. The sequenced reads were reduced to 12% when searching for UMI tags. The accuracy of the Nanopore sequencer is as low as 90% (read quality: 10) (Fig. 4), so many reads were lost, but this could be improved in future Nanopore updates with improved read quality (Luo et al. 2022). When reads with chimeric UMIs at both ends were removed, the remaining reads were reduced to 2%. Forty-nine consensus sequences were created by collecting sequences with the same UMI. These 49 consensus sequences were supposed to be unique molecules and were corrected with Nanopore reads using Medaka. The UMI tags were then removed, and the same haplotypes were merged. The 29 candidate haplotypes were obtained and annotated using the species names. Although Medaka eliminated many sequencing errors, several errors remained when comparing raw Nanopore reads. Therefore, we mapped raw Nanopore reads of other haplotypes of the same species to the most abundant Medaka-corrected consensus sequence of the species, and checked again whether other haplotypes of the species were corrected by Medaka to be the original haplotypes. Out of 29 haplotypes, 10 duplications were found, and 19 haplotypes were finally obtained (Table 2; Supplementary Table 1). Finally, 5 species (*Alectis ciliaris, Evistias acutirostris, Labracoglossa argentiventris, Parupeneus ciliates*, and *Trachurus japonicus*) were detected in 19 haplotypes. We found 5 out of 15 species (33%) for the detection sensitivity of fish species and 10 haplotypes out of 35 individuals (29%) for the detection sensitivity of populations among the detected species (Table 3). Eight of the 19 haplotypes were also confirmed by LoopSeq, another long-read sequencing method using an Illumina sequencer (Table 2). The most abundant haplotypes of each species identified by Nanopore were confirmed by LoopSeq sequenced reads. The detection of both Nanopore and LoopSeq suggested that these haplotypes were not sequencing errors or errors during the UMI correction.

**Table 2:** Analysis results of Universal HaCeD-Seq. For each of the 19 haplotypes resulting from the Universal HaCeD-Seq, the species name and the number of reads, which is the size of the cluster, are shown. The total number of all haplotypes and reads are shown in bold at the bottom of the table

Haplotype ID	Number of UMIs	Reads	D-loop length (bp)	Species	Identities (%)	Same haplotype within LoopSeq (reads of LoopSeq)
1	2	357	1092	*Alectis ciliaris*	98.7	✓(6)
2	3	3231	1056	*Evistias acutirostris*	99.9	✓(31)
3	3	4162	1040	*Labracoglossa argenteiventris*	98.9	✓(32)
4	2	3788	1040	*Labracoglossa argenteiventris*	98.7	✓(2)
5	2	1471	1040	*Labracoglossa argenteiventris*	98.8	-
6	1	1071	1039	*Labracoglossa argenteiventris*	98.9	-
7	1	826	1037	*Labracoglossa argenteiventris*	99.5	-
8	1	131	1044	*Labracoglossa argenteiventris*	97.6	-
9	1	7	1039	*Labracoglossa argenteiventris*	98.4	-
10	2	4931	1096	*Parupeneus ciliatus*	97.5	✓(2)
11	1	6311	1076	*Trachurus japonicus*	99.4	✓(35)
12	1	2220	1076	*Trachurus japonicus*	99.6	-
13	1	965	1076	*Trachurus japonicus*	99.1	-
14	1	592	1076	*Trachurus japonicus*	99.8	✓(5)
15	1	340	1076	*Trachurus japonicus*	99.4	-
16	3	334	1076	*Trachurus japonicus*	99.4	✓(56)
17	1	205	1076	*Trachurus japonicus*	99.3	-
18	1	26	1070	*Trachurus japonicus*	98.1	-
19	1	10	1070	*Trachurus japonicus*	96.3	-
Total	**29**	**30,978**

**Table 3:** Number of fish and number of haplotypes detected. A list of fish species that potentially could be detected, and a list of fish species that have been detected is shown

	Species	Number of individuals	Detected haplotypes
Fish kept in aquariums	*Alectis ciliaris*	2	1
	*Centropyge ferrugata*	4	
	*Centropyge tibicen*	1	
	*Chaetodon auripes*	1	
	*Chaetodon nippon*	3	
	*Chromis notata*	4	
	*Evistias acutirostris*	4	1
	*Labracoglossa argenteiventris*	25	7
	*Labroides dimidiatus*	1	
	*Parupeneus ciliatus*	4	1
	*Pomacanthus semicirculatus*	1	
	*Prionurus scalprum*	1	
	*Pterois lunulata*	2	
	*Thalassoma cupido*	6	
Fish fed as food	*Trachurus japonicus*	–	9

## Discussion

In this study, we designed D-loop universal primers and demonstrated a comprehensive method for estimating fish population using eDNA. We named this version of the protocol Universal HaCeD-Seq (ver. 1). We found 5 out of 15 species (33%) for the detection sensitivity of fish species and 10 haplotypes out of 35 individuals (29%) for the detection sensitivity of populations among the detected species. It has been known that the population size of Anguillids (Yoshitake et al. 2019) and *Thunnus orientalis* (Yoshitake et al. 2021) can be estimated from environmental DNA using HaCeD-Seq. This study shows the possibility of simultaneously determining the population size of multiple species by a single experiment. The MiFish primer (Miya et al. 2020), which is commonly used in environmental DNA analysis, can determine the presence or absence of a target organism, but Universal HaCeD-Seq can also provide information on the number of individuals. However, while the MiFish primers were able to detect 93.3% of the fish species in the tank (Miya et al. 2015), Universal HaCeD-Seq was only able to detect 33%. This could be because the longer eDNA fragments degrade more quickly (Jo et al. 2017). The PCR fragment length of Universal HaCeD-Seq is 1 kbp which is longer than 200 bp of MiFish primers. In the future, it is expected to improve the accuracy of the estimation of the abundance of whole fish species to combine the advantages of the species detection of MiFish primers and the population size estimation of Universal HaCeD-Seq.

We also showed that thin whole-genome sequencing can efficiently determine the full-length mitochondrial sequences of 45 species. Only the 12S rRNA gene sequences were available in public databases for *Evistias acutirostris* and *Parupeneus ciliates*, and their D-loop sequences were not known; therefore, additional mitochondrial D-loop sequences in this study made it possible to determine these species in tank 36 of the Tokyo Sea Life Park. The D-loop reference sequence should be sequenced in the future.

The mean phred quality score of the Nanopore reads was 11.9 (Fig. 4), so the average quality of the Nanopore reads was 94%, which was lower than that of the Illumina reads. Only 181,661 reads (12%) could be used for analysis using one Nanopore MinION flow cell because we eliminated 88% of the reads that did not have clear adapter sequences (Fig. 5). This is expected to improve dramatically with the use of a more accurate nanopore flow cell that will become available in the near future (Luo et al. 2022). PacBio’s HiFi sequencing is also available as a highly accurate long read sequence (Wenger et al. 2019; Patat et al. 2022), but is still relatively expensive. We also used LoopSeq as a synthetic long-read method using Illumina, but this method uses UMI tags in the kit, so it is not possible to use custom UMI tags. Therefore, errors during sequencing can be eliminated, but not during PCR using LoopSeq. Nanopore sequencing with UMI seems to be more accurate than LoopSeq because two-step PCR allows for PCR error removal using UMI tags. However, because the cost per base for nanopore sequencing is much higher than that of Illumina, a sequencing method using Illumina is desirable. However, the LoopSeq PCR Amplicon Sample Kit was discontinued in 2020 and is currently unavailable. In the future, it will be necessary to go back to BAsE-Seq (Hong et al. 2014) or other published synthetic long-read methods (Stapleton et al. 2016) which are the bases of LoopSeq, to establish a library preparation method for HaCeD-Seq.

The 1st PCR is the step of adding UMI, and only 2 cycles are ideal to add unique UMI per molecule. However, in this study, the number of cycles of 1st PCR was 5, taking into account that DNA was lost during the gel purification after 1st PCR. These additional 3 PCR cycles might increase the number of apparent molecules up to 2^3^ = 8 times. The increase in the number of apparent molecules decreases the number of reads per UMI, making it difficult to create a consensus sequence by reads derived from the same UMI. However, 29 UMIs were remained after removal of the UMI chimeras from the 49 UMIs, and 19 independent D-loop haplotypes were detected. Therefore, there were not so many chimeric UMIs, but if the 1st PCR could be performed in fewer cycles, the UMI chimeras would be suppressed, and the detection sensitivity of D-loop haplotypes would be improve.

The fish species detected in this study swim near the surface of the tank (Fig. 3). Because the sampled water was taken from the surface layer of the tank using a bucket, it is reasonable to detect many fish species near the surface layer. However, we were not able to detect fish swimming in the lower layers of the tank, and future work is needed to improve the detection sensitivity. We believe that the reason for the low detection sensitivity was the low number of valid reads (181,661 reads) (Fig. 5), which meant that fish species with low eDNA concentrations could not be detected. We expect that an increase in the number of valid reads in the future will increase the detection sensitivity and enable the detection of more species.

Fish collected from the natural environment of the sea around Japan were kept and displayed in tank 36. Because of the restriction of not being able to collect DNA from fish on display, we were unable to confirm the D-loop sequence of each individual fish, so we do not know about haplotype overlap, but seven haplotypes out of 25 fish were identified in the *Labracoglossa argentiventris*, indicating that at least seven *Labracoglossa argentiventris* were present based on eDNA. The species of fish detected in this study was the first handled in our laboratory, and the risk of contamination is considered low because mitochondria were sequenced to determine the reference sequence after eDNA sequencings were performed. This method has promise as a method for determining the lower limit of the number of individuals present in the environment, which is not possible with conventional eDNA research methods that target 12S rRNA genes. As data on D-loop haplotype diversity for each fish species becomes available, it will be possible to estimate the number of individuals more accurately from the number of haplotypes detected, taking into account the degree of overlap.

This study demonstrates the possibility of comprehensively obtaining information related to population size from eDNA. In the future, we believe that this method can be used in combination with 12S rRNA-based survey methods, such as the MiFish primer, which has high detection sensitivity, to greatly improve the accuracy of estimating methods of fish resources that are currently highly dependent on fishing catches.

## Supplementary Information

Below is the link to the electronic supplementary material.

Supplementary file1 (PPTX 4357 KB)

Supplementary file2 (STL 53 KB)

Supplementary file3 (XLSX 12 KB)

## Data Availability

All sequencing data were registered in the NCBI SRA database under the accession number PRJNA771544. The mitochondrial sequences were registered under the following accession numbers: OK554506-OK554545.

## References

[CR1] Camacho C, Coulouris G, Avagyan V (2009). BLAST+: architecture and applications. BMC Bioinforma.

[CR2] Cheng Y-Z, Xu T-J, Jin X-X (2012). Universal primers for amplification of the complete mitochondrial control region in marine fish species. Mol Biol.

[CR3] Clare EL, Economou CK, Faulkes CG (2021). eDNAir: proof of concept that animal DNA can be collected from air sampling. PeerJ.

[CR4] Crooks GE, Hon G, Chandonia J-M, Brenner SE (2004). WebLogo: a sequence logo generator. Genome Res.

[CR5] Edgar RC (2004). MUSCLE: a multiple sequence alignment method with reduced time and space complexity. BMC Bioinforma.

[CR6] Fan X, Yan T, Hou T (2022). Mitochondrial changes in fish cells in vitro in response to serum deprivation. Fish Physiol Biochem.

[CR7] Garlapati D, Charankumar B, Ramu K (2019). A review on the applications and recent advances in environmental DNA (eDNA) metagenomics. Rev Environ Sci Biotechnol.

[CR8] Hartmann N, Reichwald K, Wittig I (2011). Mitochondrial DNA copy number and function decrease with age in the short-lived fish Nothobranchius furzeri. Aging Cell.

[CR9] Hong LZ, Hong S, Wong HT (2014). BAsE-Seq: a method for obtaining long viral haplotypes from short sequence reads. Genome Biol.

[CR10] Ichinokawa M, Okamura H, Kurota H (2017). The status of Japanese fisheries relative to fisheries around the world. ICES J Mar Sci.

[CR11] Ishikawa S, Aoyama J, Tsukamoto K, Nishida M (2001). Population structure of the Japanese eel *Anguilla japonica* as examined by mitochondrial DNA sequencing. Fish Sci.

[CR12] Iwasaki W, Fukunaga T, Isagozawa R (2013). MitoFish and MitoAnnotator: a mitochondrial genome database of fish with an accurate and automatic annotation pipeline. Mol Biol Evol.

[CR13] Jane SF, Wilcox TM, McKelvey KS (2015). Distance, flow and PCR inhibition: eDNA dynamics in two headwater streams. Mol Ecol Resour.

[CR14] Jo T, Murakami H, Masuda R (2017). Rapid degradation of longer DNA fragments enables the improved estimation of distribution and biomass using environmental DNA. Mol Ecol Resour.

[CR15] Karst SM, Ziels RM, Kirkegaard RH (2021). High-accuracy long-read amplicon sequences using unique molecular identifiers with nanopore or PacBio sequencing. Nat Methods.

[CR16] Katoh K, Misawa K, Kuma K, Miyata T (2002). MAFFT: a novel method for rapid multiple sequence alignment based on fast Fourier transform. Nucleic Acids Res.

[CR17] Knudsen SW, Ebert RB, Hesselsøe M (2019). Species-specific detection and quantification of environmental DNA from marine fishes in the Baltic Sea. J Exp Mar Biol Ecol.

[CR18] Kumar G, Kocour M, Kunal SP (2016). Mitochondrial DNA variation and phylogenetic relationships among five tuna species based on sequencing of D-loop region. Mitochondrial DNA Part A.

[CR19] Li D, Liu C-M, Luo R (2015). MEGAHIT: an ultra-fast single-node solution for large and complex metagenomics assembly via succinct de Bruijn graph. Bioinformatics.

[CR20] Luo J, Meng Z, Xu X (2022). Systematic benchmarking of nanopore Q20+ kit in SARS-CoV-2 whole genome sequencing. Front Microbiol.

[CR21] Maruyama A, Nakamura K, Yamanaka H (2014). The release rate of environmental DNA from juvenile and adult fish. PLOS ONE.

[CR22] Miya M, Sato Y, Fukunaga T (2015). MiFish, a set of universal PCR primers for metabarcoding environmental DNA from fishes: detection of more than 230 subtropical marine species. R Soc Open Sci.

[CR23] Miya M, Gotoh RO, Sado T (2020). MiFish metabarcoding: a high-throughput approach for simultaneous detection of multiple fish species from environmental DNA and other samples. Fish Sci.

[CR24] Nomura S, Kobayashi T, Agawa Y (2014). Genetic population structure of the Pacific bluefin tuna Thunnus orientalis and the yellowfin tuna Thunnus albacares in the North Pacific Ocean. Fish Sci.

[CR25] Okabe T, Suguro N, Koito T (2020). Genetic and morphological characteristics in the local population of the landlocked salmon Oncorhynchus masou originally distributed in Kanagawa Prefecture, Japan. Mar Biotechnol.

[CR26] Patat AS, Sen F, Erdogdu BS (2022). Construction and characterization of a de novo draft genome of garden cress (Lepidium sativum L.). Funct Integr Genomics.

[CR27] Rees HC, Maddison BC, Middleditch DJ (2014). REVIEW: the detection of aquatic animal species using environmental DNA – a review of eDNA as a survey tool in ecology. J Appl Ecol.

[CR28] Ruppert KM, Kline RJ, Rahman MS (2019). Past, present, and future perspectives of environmental DNA (eDNA) metabarcoding: a systematic review in methods, monitoring, and applications of global eDNA. Glob Ecol Conserv.

[CR29] Salter I, Joensen M, Kristiansen R (2019). Environmental DNA concentrations are correlated with regional biomass of Atlantic cod in oceanic waters. Commun Biol.

[CR30] Sang TK, Chang HY, Chen CT, Hui CF (1994). Population structure of the Japanese eel, Anguilla japonica. Mol Biol Evol.

[CR31] Song N, Jia N, Yanagimoto T (2013). Genetic differentiation of Trachurus japonicus from the Northwestern Pacific based on the mitochondrial DNA control region. Mitochondrial DNA.

[CR32] Stapleton JA, Kim J, Hamilton JP (2016). Haplotype-phased synthetic long reads from short-read sequencing. PLOS ONE.

[CR33] Stoeckle MY, Adolf J, Charlop-Powers Z (2020). Trawl and eDNA assessment of marine fish diversity, seasonality, and relative abundance in coastal New Jersey.

[CR34] Thomsen PF, Willerslev E (2015). Environmental DNA – an emerging tool in conservation for monitoring past and present biodiversity. Biol Conserv.

[CR35] Tzeng T-D (2007) Population Structure and historical demography of the spotted mackerel (Scomber australasicus) off Taiwan inferred from mitochondrial control region sequencing. Zool Stud 8

[CR36] Wenger AM, Peluso P, Rowell WJ (2019). Accurate circular consensus long-read sequencing improves variant detection and assembly of a human genome. Nat Biotechnol.

[CR37] Yoshitake K, Yoshinaga T, Tanaka C (2019). HaCeD-Seq: a novel method for reliable and easy estimation about the fish population using haplotype count from eDNA. Mar Biotechnol N Y N.

[CR38] Yoshitake K, Fujiwara A, Matsuura A (2021). Estimation of tuna population by the improved analytical pipeline of unique molecular identifier-assisted HaCeD-Seq (haplotype count from eDNA). Sci Rep.

[CR39] Zhu Y, Cheng Q, Rogers SM (2016). Genetic structure of Scomber japonicus (Perciformes: Scombridae) along the coast of China revealed by complete mitochondrial cytochrome b sequences. Mitochondrial DNA Part DNA Mapp Seq Anal.

