# Supplementary material for: Pilot study of a comprehensive resource estimation method from environmental DNA using universal D-loop amplification primers
Source: Funct Integr Genomics. 2023 Mar 22;23(2):96. doi: 10.1007/s10142-023-01013-3 (PMC10033627; doi:10.1007/s10142-023-01013-3)
Supplement: Supplementary file 1 — Supplementary file1 (PPTX 4357 KB) [file 10142_2023_1013_MOESM1_ESM.pptx]

## Slide 1
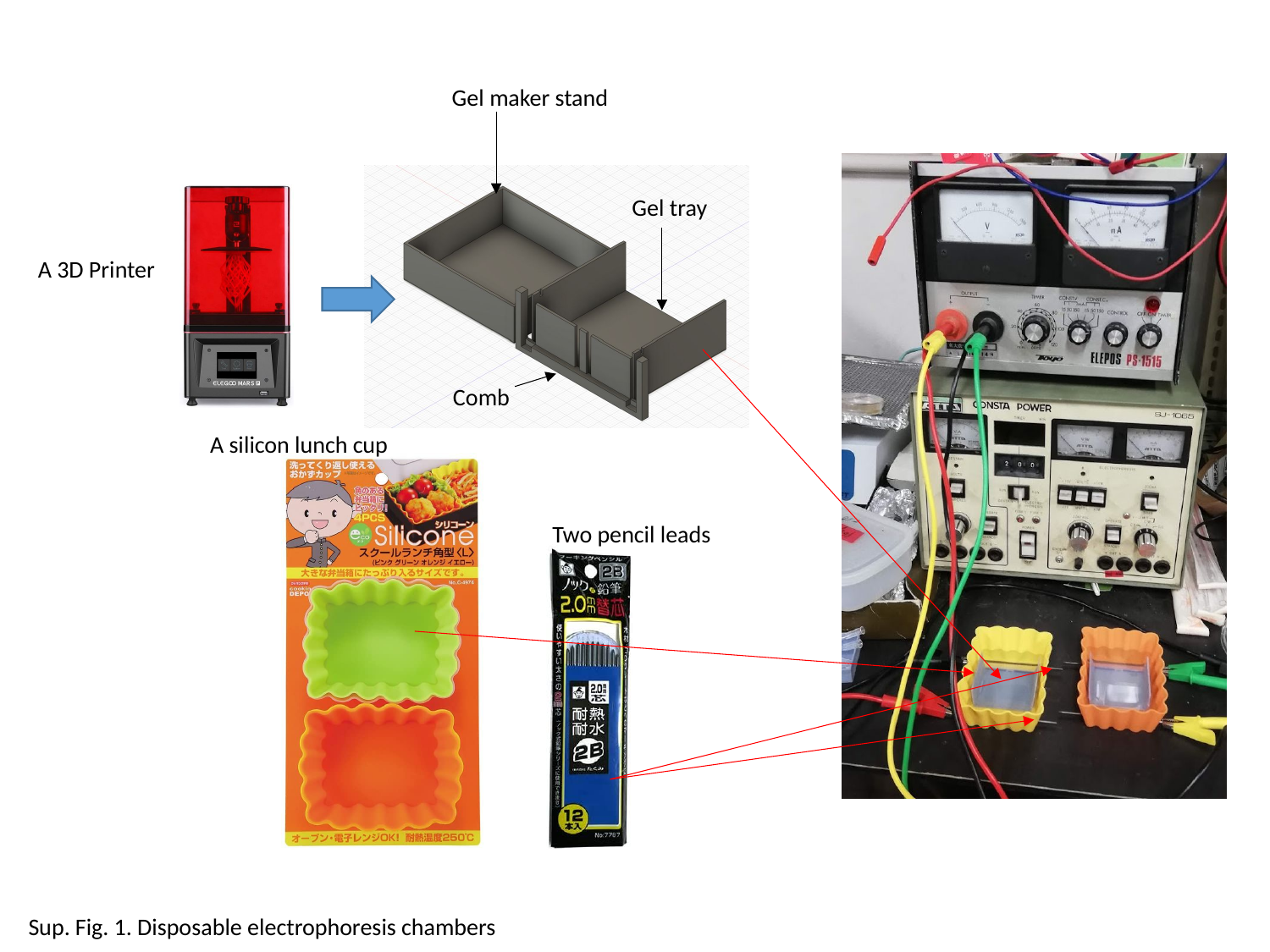

Gel maker stand
Gel tray
A 3D Printer
Comb
A silicon lunch cup
Two pencil leads
Sup. Fig. 1. Disposable electrophoresis chambers

## Slide 2
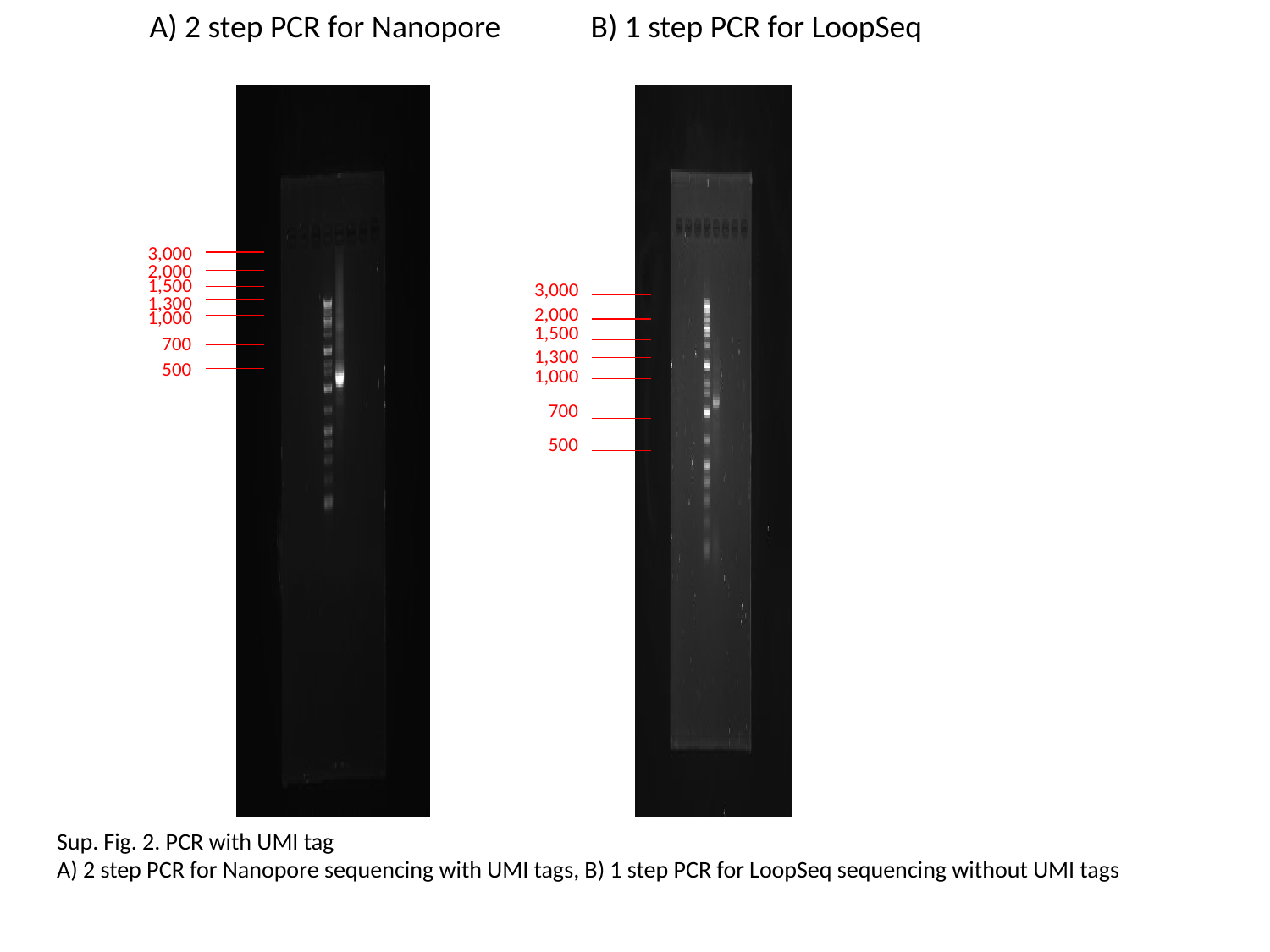

A) 2 step PCR for Nanopore
B) 1 step PCR for LoopSeq
3,000
2,000
1,500
3,000
1,300
2,000
1,000
1,500
700
1,300
500
1,000
700
500
Sup. Fig. 2. PCR with UMI tag
A) 2 step PCR for Nanopore sequencing with UMI tags, B) 1 step PCR for LoopSeq sequencing without UMI tags
